# The CDK-Activating Kinase (CAK) Csk1 Is Required for Normal Levels of Homologous Recombination and Resistance to DNA Damage in Fission Yeast

**DOI:** 10.1371/journal.pone.0001492

**Published:** 2008-01-30

**Authors:** Hilary B. Gerber, Yana Pikman, Robert P. Fisher

**Affiliations:** 1 Molecular Biology Program, Memorial Sloan-Kettering Cancer Center, New York, New York, United States of America; 2 Programs in Biochemistry, Cell and Molecular Biology, Cornell University Graduate School of Medical Sciences, New York, New York, United States of America; National Institute on Aging, United States of America

## Abstract

**Background:**

Cyclin-dependent kinases (CDKs) perform essential roles in cell division and gene expression in all eukaryotes. The requirement for an upstream CDK-activating kinase (CAK) is also universally conserved, but the fission yeast *Schizosaccharomyces pombe* appears to be unique in having two CAKs with both overlapping and specialized functions that can be dissected genetically. The Mcs6 complex—orthologous to metazoan Cdk7/cyclin H/Mat1—activates the cell-cycle CDK, Cdk1, but its non-redundant essential function appears to be in regulation of gene expression, as part of transcription factor TFIIH. The other CAK is Csk1, an ortholog of budding yeast Cak1, which activates all three essential CDKs in *S. pombe*—Cdk1, Mcs6 and Cdk9, the catalytic subunit of positive transcription elongation factor b (P-TEFb)—but is not itself essential.

**Methodology/Principal Findings:**

Cells lacking *csk1^+^* are viable but hypersensitive to agents that damage DNA or block replication. Csk1 is required for normal levels of homologous recombination (HR), and interacts genetically with components of the HR pathway. Tests of damage sensitivity in *csk1*, *mcs6* and *cdk9* mutants indicate that Csk1 acts pleiotropically, through Cdk9 and at least one other target (but not through Mcs6) to preserve genomic integrity.

**Conclusions/Significance:**

The two CAKs in fission yeast, which differ with respect to their substrate range and preferences for monomeric CDKs versus CDK/cyclin complexes as substrates, also support different functions of the CDK network in vivo. Csk1 plays a non-redundant role in safeguarding genomic integrity. We propose that specialized activation pathways dependent on different CAKs might insulate CDK functions important in DNA damage responses from those capable of triggering mitosis.

## Introduction

A network of cyclin-dependent kinases (CDKs) coordinates eukaryotic cell division with duplication, maintenance and regulated expression of the genome. To attain full activity, CDKs require cyclin-binding and phosphorylation within the activation segment (T-loop) by a CDK-activating kinase (CAK) [1]. In metazoans, the CAK is itself a CDK, the heterotrimeric Cdk7/cyclin H/Mat1 complex, which is also a component of the general transcription factor TFIIH [reviewed in [2]]. In the budding yeast *Saccharomyces cerevisiae*, however, the Cdk7 ortholog Kin28 is a dedicated component of the transcription machinery with no CAK activity [3], [4]. CAK function instead resides in Cak1, a monomeric kinase only distantly related to CDKs [5]–[7]. The fission yeast *Schizosaccharomyces pombe* has two CAKs: 1) the Mcs6/Mcs2/Pmh1 complex, orthologous to Cdk7/cyclin H/Mat1 [8]–[12]; and 2) Csk1, an ortholog of Cak1 [12]–[14].

*S. pombe* affords a unique opportunity to dissect functions of the CDK network genetically, with the goal of understanding the coordination of cell division, growth and the DNA damage response in all eukaryotes. Separate ablation of each of the two CAKs in fission yeast can reveal how signaling through CDKs is coordinated and how different subpathways are insulated from one another. The Mcs6 complex is essential, but that might be because of a role in gene expression [11]; its ability to activate Cdk1 (the major cell cycle CDK) appears to be redundant with that of Csk1 [12], [15]. Components of the Mcs6 complex were initially isolated in genetic screens for regulators of the G2/M transition [14], [16], and it has been suggested that the CAK activity of Csk1 cannot normally support mitotic entry [17]. Phenotypic analysis of multiple *mcs6*, *mcs2* and *pmh1* mutants, however, failed to uncover a non-redundant role for the complex in activating Cdk1 or promoting mitosis [8]–[11], [14], [15]. Csk1, although not essential for viability, is required for growth in suboptimal conditions or in the absence of normal Mcs6 complex function. It is a general CAK that activates Cdk1, Mcs6 and Cdk9—an essential homolog of positive transcription elongation factor b (P-TEFb) in metazoans [12], [15], [18]–[20].

When proliferating cells encounter DNA damage, they must pause cell cycle progression in order to allow repair of the lesion(s), if possible [21]. In fission yeast and metazoans, the normal checkpoint response to damage during G2 is a cell-cycle arrest due to inhibition of CDK [reviewed in [22]]. How repair or signaling pathways that depend on the *activity* of CDKs [23]–[25] can operate under these conditions remains a mystery. One possible solution to this paradox is the existence of multiple CDK activation pathways that can be insulated from one another, to permit proper control of the DNA damage response without triggering mitosis prematurely. We sought to determine whether the presence in *S. pombe* of two distinct CDK-activating enzymes might provide such insulation.

Here we show that strains lacking *csk1^+^* are hypersensitive to DNA-damaging agents and defective in homologous recombination (HR), suggesting that normal CDK activation—by a full complement of CAKs—is needed to maintain genomic integrity. A hypomorphic *cdk9* mutant in which the kinase is refractory to activation by Csk1 was also hypersensitive to DNA damage, indicating a role for Cdk9, possibly dependent on activating phosphorylation, in the normal DNA damage response. A T-loop mutation that activates Cdk9 constitutively, however, did not suppress UV-hypersensitivity of a *csk1Δ* strain. Bypassing the CAK requirement for Cdk9 thus uncovered Csk1-dependent functions presumably mediated by other CDKs. Loss of the non-essential, single-subunit CAK therefore has pleiotropic effects on the fission yeast CDK network and its functions in response to DNA damage.

## Results

### Csk1 is required for normal resistance to DNA damage

Only when both Mcs6 and Csk1 are compromised do fission yeast cells arrest their division cycle due to insufficient CAK activity [12]. In an *mcs6-13 csk1Δ* strain, progression through both G1/S and G2/M transitions was blocked at restrictive temperature. Phosphorylation of Cdk1 on T-loop residue Thr-167 decreased whereas phosphorylation of another Mcs6 target—the carboxyl-terminal domain (CTD) of the RNA Polymerase (Pol) II large subunit—was maintained, suggesting that inactivation of Mcs6 in this setting was incomplete and preferentially affected CAK function [15]. Tight temperature-sensitive mutations in *mcs6* or *pmh1* arrested at a different point in the cell cycle with a hypophosphorylated CTD but near-normal levels of Cdk1 T-loop phosphorylation [11], [15].

**Figure 1:**
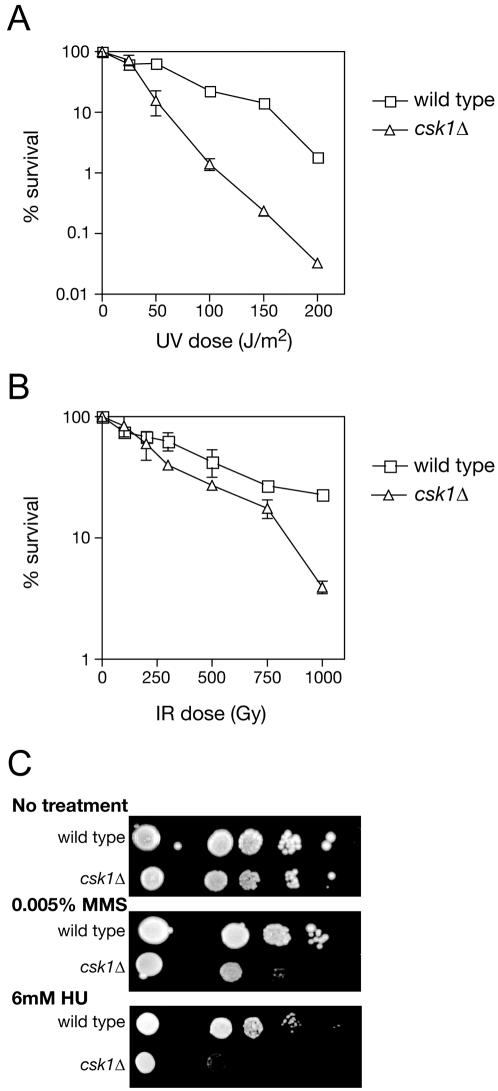
A *csk1Δ* strain is hypersensitive to DNA damaging agents. Survival was measured after irradiation with UV (A) or IR (B) of the following strains: wild type (JS78), *csk1Δ* (JS155). (C) 10-fold serial dilutions of the wild-type and *csk1Δ* strains [as in (A)] in mid-log phase were plated on fresh media containing no drug (top), 0.005% MMS (middle) or 6 mM hydroxyurea (bottom), and incubated 3–5 days before photographing.

Just as functional overlap between the two CAKs helped reveal essential functions of Mcs6 in gene expression, it allowed detection of specific defects in cells living without Csk1. A *csk1Δ* strain was more sensitive than a wild-type strain to killing by ultraviolet light (UV; Fig. 1A) and, to a lesser extent, ionizing radiation (IR; Fig. 1B). In addition, growth of *csk1Δ* cells was more severely impaired by continuous exposure to the alkylating agent methylmethanesulfonate (MMS) and the replication inhibitor hydroxyurea (HU) than that of wild-type strains (Fig. 1C). Similarly, hypersensitivity of a *csk1Δ* strain to HU and the DNA-damaging agents ethylmethanesulfonate and bleomycin was detected in a systematic deletion analysis of fission yeast genes encoding protein kinases [18]. Together, the impaired survival and growth of *csk1Δ* cells under a variety of conditions suggest a generalized hypersensitivity to genotoxic stress.

**Figure 2:**
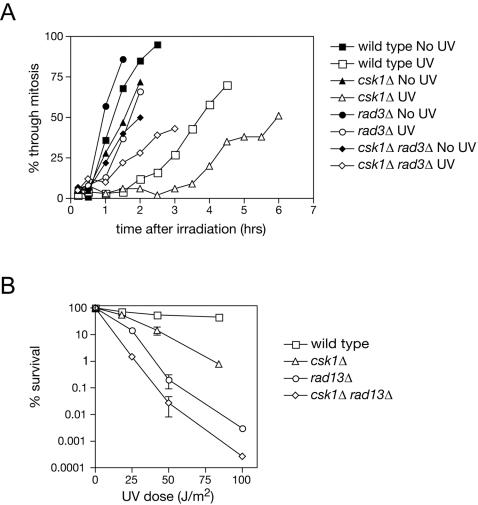
Csk1 is not required for activation of the DNA damage checkpoint or for NER. (A) WT (JS78), *csk1Δ* (JS155), *rad3Δ* (YP46) and *csk1Δ rad3Δ* (YP68) cells were synchronized in G2 by fractionation in lactose gradients and irradiated with 40 J/m^2^ UV light. Samples were taken every 30 min and the percent of cells passing through mitosis was measured by counting binucleated cells, septated cells and doublets. (B) Survival after UV irradiation of the following strains: wild type (JS78), *csk1Δ* (JS155), *rad13Δ* (YP1), *csk1Δ rad13Δ* (YP85).

Increased sensitivity to DNA lesions could be due to a defect in damage repair or in the cellular response to damage, i.e. the proper function of the DNA damage checkpoint [21], [22]. To test the latter possibility, we monitored checkpoint integrity in *csk1Δ* cells exposed to DNA damage. We obtained cells synchronized in G2 by centrifugation in lactose gradients, irradiated them with 40 J/m^2^ UV light and recorded the total number of cells that passed through mitosis at 30-min intervals (Fig. 2A). Unlike a *rad3Δ* strain that lacks the checkpoint kinase (and ATR/ATM homolog) Rad3 [26], the *csk1Δ* mutant could arrest the cell cycle in response to UV irradiation, and that arrest depended on *rad3^+^*. However, re-entry into the cell cycle was slower and less synchronous in *csk1Δ* than in wild type cells, suggesting a defect in DNA damage repair and/or checkpoint recovery.

### The NER pathway is functional in the absence of Csk1

The differential effects of UV and IR on survival of the *csk1Δ* strain (Fig. 1A, B) indicate a specific problem in handling DNA damage caused by the former, and suggest a defect in repair. The major pathway for removing UV-induced lesions is nucleotide excision repair (NER) [27], in which TFIIH plays an essential role [28], [29]. We therefore investigated a possible function for Csk1 (and perhaps, by extension, its target the Mcs6 complex) in NER, by testing for genetic epistasis with a known component of the pathway. Deletion of the *rad13* gene abolishes NER [27], but a *rad13Δ csk1Δ* double mutant strain was more UV-sensitive than either the *csk1Δ* or *rad13Δ* single mutant parent (Fig. 2B). Therefore NER confers UV-resistance in the absence of Csk1, suggesting a role for the CAK in another repair pathway.

### Interactions between *csk1* and genes in the homologous recombination pathway

Homologous Recombination (HR) is a primary mechanism by which yeast cells repair double strand breaks (DSBs) in DNA [reviewed in [30]]. Fission yeast mutants defective in HR, unlike their counterparts in budding yeast, are very sensitive to killing by UV light [27]. Rhp51, Rad51 and the homologous bacterial RecA protein play a conserved role at a DSB, forming a nucleoprotein filament on single-stranded DNA (ssDNA), which can invade a double-stranded region on a sister or homolog [31]. Rhp54/Rad54 assists in the process, as does a complex of Rhp55 and Rhp57 (Rad55 and Rad57 in budding yeast), paralogs of Rad51 [31], [32]. In *S. cerevisiae*, Rad55/57 acts in Rad51 filament assembly, whereas Rad54 acts later, during strand invasion [33].

**Figure 3:**
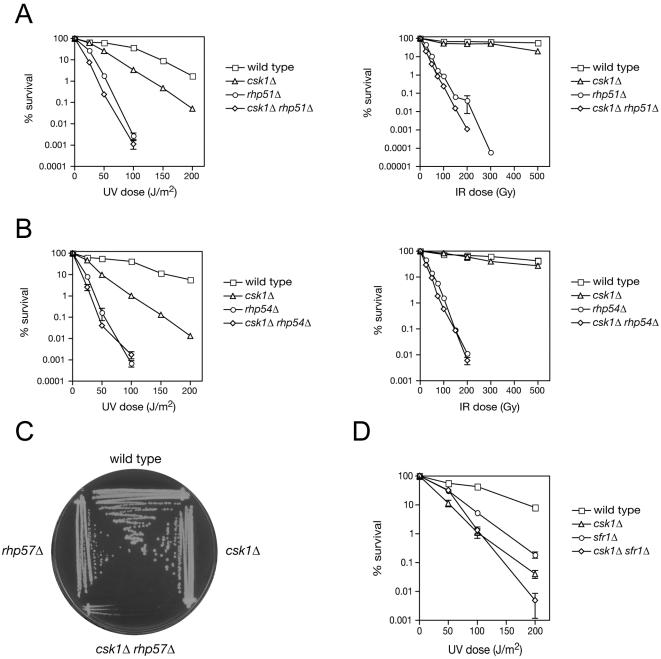
Epistasis analysis with homologous recombination genes. (A) Survival after irradiation with UV or IR of the strains: (A) wild type (JS78), *csk1Δ* (JS155), *rhp51Δ* (YP6), *csk1Δ rhp51Δ* (HD4-6); (B) wild type (JS78), *csk1Δ* (JS155), *rhp54Δ* (YP25), *csk1Δ rhp54Δ* (HD2-55). (C) Cells were streaked onto YES plates and incubated for 4 days at 30°C before being photographed. Strains: wild type (JS78), *csk1Δ* (JS155), *rhp57Δ* (YP27), *csk1Δ rhp57Δ* (HG123). (D) Survival after UV irradiation of the strains: wild type (JS78), *csk1Δ* (JS155), *sfr1Δ* (HG24), *csk1Δ sfr1Δ* (HG31).

We tested if *csk1* interacts with genes important for HR repair by epistasis analysis. The *rhp51Δ csk1Δ* double mutant was very slightly more sensitive to both UV and IR than was the *rhp51Δ* single mutant (Fig. 3A), whereas *rhp54Δ* was clearly epistatic to *csk1Δ* (Fig. 3B), suggesting a role for Csk1 in the HR repair pathway. In contrast, we observed a synthetic interaction between *csk1Δ* and *rhp57Δ*. The *csk1Δ rhp57Δ* double mutants grew very slowly (Fig. 3C and data not shown), suggesting compromised genomic stability even in unchallenged cells.

The severe effect on growth in the absence of DNA damaging agents suggests a requirement for either Csk1 or Rhp57 in order to deal with DSBs that occur normally, perhaps during replication. Taken together, the genetic data suggest that Csk1 acts upstream of Rhp51 and Rhp54, in a pathway separate from the one including Rhp55/57, to promote HR. Such a pathway has been described in *S. pombe*, dependent on the heterodimeric Swi5/Sfr1 complex [34]–[36]. We observed complex genetic interactions between *csk1* and *sfr1* (Fig. 3D): at low UV doses, deletion of *sfr1^+^* suppressed hypersensitivity due to *csk1Δ*; at an intermediate dose, *csk1Δ* was epistatic to *sfr1Δ*; and at high doses, there was an additive effect on sensitivity (see Discussion for a possible interpretation).

### Csk1 is required for normal frequency of HR

**Figure 4:**
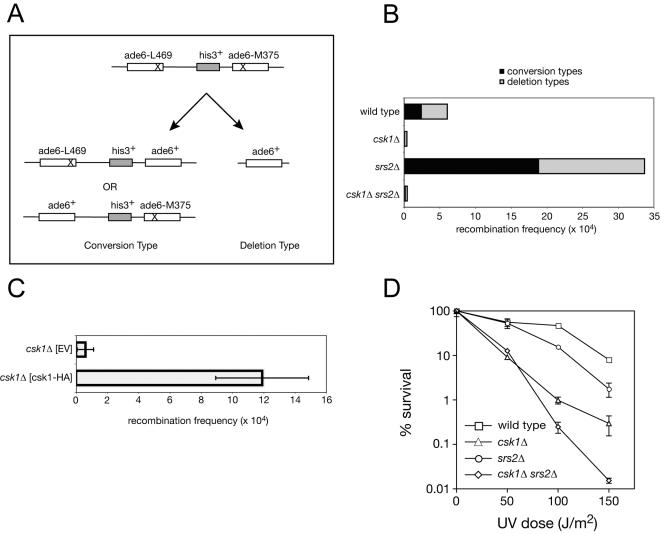
Loss of Csk1 impairs HR. (A) Schematic of tandem *ade6* alleles used to determine spontaneous recombination frequencies. Conversion-type recombination events result in his+ ade+ colonies and deletion-type recombination events result in his- ade+ colonies. (B) Recombination frequencies (strain: conversion type±standard deviation, deletion type±standard deviation): wild type (HG11: 2.4±0.8, 3.7±1.1); *csk1Δ* (HG16: 0.09±0.13, 0.30±0.28); *srs2Δ* (HG13: 19.3±9.2, 14.4±3.9); *csk1Δ srs2Δ* (HG19: 0.14±0.08, 0.30±0.22). (C) Rescue of *csk1Δ* hypo-recombination with overexpression of *csk1^+^* (strain: total recombination frequency±standard deviation): *csk1Δ [EV]* (HG142: 0.60±0.52); *csk1Δ [csk1^+^]* (HG144: 11.9±3.0). (D) Survival after UV irradiation of the following strains: wild type (JS78), *csk1Δ* (JS155), *srs2Δ* (HG119), *csk1Δ srs2Δ* (HG112).

In order to test whether Csk1 promotes HR in unperturbed cells, we measured the frequency of *ade^+^* colonies arising by spontaneous recombination between two different, non-functional *ade6* mutant alleles flanking a *his^+^* gene [the assay system is described in [37]]. Histidine prototrophy is preserved in conversion-type, and lost in deletion-type, recombinants (Fig. 4A). Overall, spontaneous recombination frequencies were reduced by 94% in a strain lacking *csk1^+^* compared to a wild-type strain (0.39±0.41 for *csk1Δ* vs. 6.1±1.9 for wild type) and the percentage of conversion types was also reduced (23% of total HR events for *csk1Δ* compared to 39% for wild type) (Fig. 4B). In *rhp57Δ* or *rhp51Δ* strains, in contrast, total HR frequencies are elevated and skewed heavily towards the deletion type [38]. The low frequency of both conversion and deletion indicates impairment of both Rhp51-dependent and –independent HR in *csk1Δ* strains. Near-normal HR frequency was restored in the *csk1Δ* strain by *csk1^+^* expressed from a plasmid (Fig. 4C).

We also asked if *csk1^+^* and *srs2^+^* have opposite effects on HR in vivo. The *srs2^+^* gene encodes a DNA helicase that negatively regulates recombination [38]–[40]. In maintaining viability after UV irradiation (Fig. 4D), *csk1Δ* was epistatic to *srs2Δ* at low doses, but the two mutations caused synthetic hypersensitivity at higher doses (see Discussion). In budding yeast, Srs2 suppresses recombination leading to crossovers [41] through its ability to disrupt Rad51-ssDNA filaments [40], [42], [43]; deletion of *srs2^+^* in *S. pombe* causes elevated levels of spontaneous HR, presumably by a similar mechanism [38]. Deletion of *csk1^+^* suppressed hyper-recombination in an *srs2Δ* strain (Fig. 4B), consistent with Csk1 working in opposition to Srs2, possibly to promote Rhp51 function.

### Csk1 functions in the DNA damage response through multiple targets

**Figure 5:**
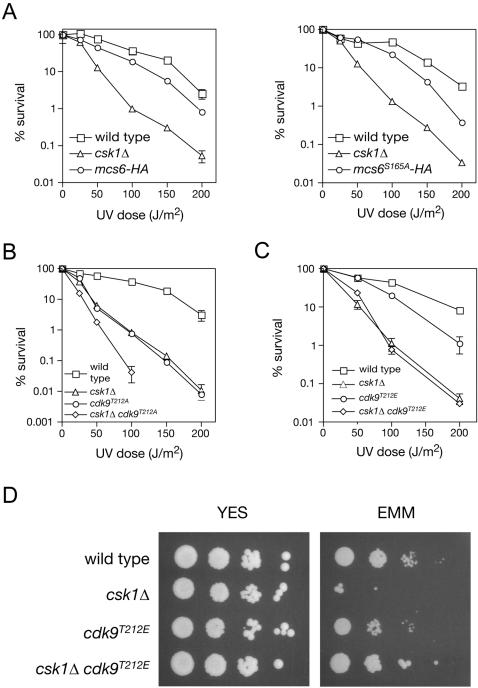
Multiple Csk1 targets contribute to the *csk1Δ* UV-sensitivity and growth phenotypes. Survival after UV irradiation of the following strains: (A) wild type (JS78), *csk1Δ* (JS155), *mcs6-HA* (JS167), *mcs6^S165A^-HA* (JS207); (B) wild type (JS78), *csk1Δ* (JS155), *cdk9^T212A^* (HD7-24), *csk1Δ cdk9^T212A^* (HD7-44); and (C) wild type (JS78), *csk1Δ* (JS155), *cdk9^T212E^* (HG127), *csk1Δ cdk9^T212E^* (HG133). (D) Spot assays on YES and EMM plates with the following strains: wild type (JS78), *csk1Δ* (JS155), *cdk9^T212E^* (HG127), *csk1Δ cdk9^T212E^* (HG133).

Csk1 promotes survival of DNA damage, presumably through one or more downstream CDKs. To ascertain which kinase(s) might require activation by Csk1 to perform a function in the DNA damage response, we mutated the sites phosphorylated by Csk1 in two essential *S. pombe* CDKs, Mcs6 and Cdk9, and tested the resulting mutants for DNA damage-sensitivity. The addition of a hemagglutinin (HA) epitope to the carboxyl terminus of Mcs6 caused a mild UV-hypersensitivity phenotype, which was not further exacerbated by mutation of the T-loop phosphorylation site, Ser165, to Ala; the *mcs6^S165A^-HA* strain had a UV sensitivity similar to that of tagged but otherwise wild-type *mcs6-HA* (Fig. 5A). This suggests the *mcs6^S165A^* mutation by itself did not increase UV-sensitivity, and that failure to activate Mcs6 fully is unlikely to contribute to the DNA damage-sensitivity of a *csk1Δ* strain.

In contrast, mutation of the Csk1 target site Cdk9-Thr212 [20] to Ala caused UV-hypersensitivity similar to that of a *csk1Δ* strain (Fig. 5B), suggesting a role for the activating phosphorylation of Cdk9 in the DNA damage response. However, a *csk1Δ cdk9^T212A^* strain was more sensitive than either single-mutant parent. We previously observed synthetic growth defects in this strain, indicating that the T-loop mutation impairs physiologic Cdk9 function beyond the simple ∼10-fold reduction in kinase activity due to the absence of activating phosphorylation [20]. The synthetic effect on UV-sensitivity likewise implies that some radiation-induced lethality was caused by the amino-acid substitution per se, independent of phosphorylation. Conversely, because the exacerbation of sensitivity by loss of Csk1 in a *cdk9^T212A^* background cannot be due to diminished function of the Cdk9 protein, Csk1 must have at least one other target in a DNA repair pathway.

The *cdk9^T212A^* mutation rendered the kinase insensitive to stimulation by Csk1 in vitro, and phenocopied *csk1Δ* in both its cold-sensitivity and poor growth on the minimal medium EMM [20]. In contrast, a *cdk9^T212E^* mutation mimicked constitutive T-loop phosphorylation, resulting in a CAK-independent kinase with nearly wild-type activity in vitro [20]. In vivo, a *cdk9^T212E^* strain was slightly more UV-sensitive than a wild-type strain (Fig. 5C). This strain grew normally on minimal media, and the *cdk9^T212E^* mutation suppressed poor growth on EMM due to *csk1Δ* (Fig. 5D). The *csk1Δ cdk9^T212E^* double mutant was just as UV-sensitive as a *csk1Δ* single mutant, however (Fig. 5C), indicating that: 1) functions of Csk1 and Cdk9 in growth and DNA damage responses are genetically separable; and 2) much of the UV-sensitivity in a *csk1Δ* strain is due to impaired function of another CAK target, such as Cdk1 (encoded by *cdc2^+^*), which is known to regulate HR in both budding and fission yeast [23], [44], [45].

## Discussion

We have uncovered a role for CAK in the DNA damage response. Mutants with defects in the Mcs6 complex—the essential CAK in *S. pombe*, which also participates in the control of transcription by RNA Pol II [2], [11]—are not as UV-sensitive as *csk1Δ* strains (Fig. 5 and data not shown), suggesting a specialized function for Csk1, not redundant with Mcs6, in protecting genome integrity. There are several possible explanations for this specificity, which are not mutually exclusive.

Csk1 is a general CAK able to activate all fission yeast and mammalian CDKs with which it has been tested, whereas Mcs6 can only activate *S. pombe* Cdk1 [12], [19], [20]. The specific requirement for Csk1 in response to DNA damage could—and, to a degree, probably does—reflect its unique ability to activate Cdk9 [20]. The epistatic relationship between *csk1Δ* and *cdk9^T212E^*, however, implicates additional Csk1 targets. Mcs6, the other essential CDK activated by Csk1, requires T-loop phosphorylation for full activity [12], [19], but preventing that phosphorylation did not impair survival of UV-irradiation. The CDK/cyclin complex Lsk1/Lsc1 is a second, non-essential P-TEFb homolog in *S. pombe* [46]. Deletion of *lsk1* produced sensitivities to HU and DNA-damaging agents similar to those caused by *csk1Δ* [18]. The budding yeast ortholog of Lsk1 is Ctk1, which is activated in vivo by Cak1 [47]. Lsk1 is therefore another likely (and possibly exclusive) target of Csk1, likely to be important for normal resistance to DNA damage.

Csk1-dependent functions in the DNA damage response need not be restricted to ones performed by its exclusive targets. Csk1 and Mcs6 can both activate Cdk1 in vitro [12] and are likely to do so in vivo [15], raising the possibility of two separate activation pathways to support distinct Cdk1-dependent functions. Insulation of those pathways could be achieved temporally, spatially or kinetically. Neither Mcs6/Mcs2 nor Csk1 fluctuates significantly with respect to activity during the cell cycle [14], [19], and neither CAK appears to discriminate between Cdk1 complexes containing interphase or mitotic cyclins [12], making simple temporal regulation seem unlikely. Specific regulation of either Mcs6 or Csk1 in response to damaged DNA or stalled replication remains to be investigated. Spatial separation of CDK activation pathways is another possibility. Cak1 in budding yeast is predominantly cytoplasmic [48], whereas Cdk7 in higher eukaryotes is largely nuclear [49], [50], although it has also been reported to shuttle between nucleus and cytoplasm [51].

Another potential explanation of the specific requirement for Csk1 is kinetically distinct activation pathways driven by the two types of CAK. In vitro, the Cdk7 and Cak1/Csk1 classes are distinguished by their substrate preferences. Human Cdk7 recognizes the mitotic CDK, Cdk1, only in a complex with cyclin [52], whereas Cak1 and Csk1 prefer CDK monomers [53], [54]. In budding yeast, the cell-cycle CDK (Cdk1, product of the *CDC28* gene) is phosphorylated on its T-loop in vivo while in monomeric form, and throughout the cell cycle [55]. Co-expression of *S. pombe* Csk1 and Cdk1 in insect cells likewise generated monomeric Cdk1 that was phosphorylated on Thr167 and could be activated by cyclin in a single step in vitro [15]. A similar pathway operating in fission yeast could generate active CDK even in cells arrested in response to DNA damage, because the inhibitory kinases that phosphorylate Tyr15 of Cdk1—and which are terminal effectors of negative signaling by the G2 checkpoint—act preferentially on CDK/cyclin complexes [56], [57].

Tracing the connections between Csk1 and defined repair pathways through individual CDK intermediaries is difficult, because CAK function is pleiotropic. A case in point is the unexpected involvement of Cdk9 in the response to UV-induced damage. Bypassing the requirement for T-loop phosphorylation to activate Cdk9, in *cdk9^T212E^* mutant strains, could permit a more direct test of Cdk1's role, but might also uncover added complexity: e.g. functions of other, as-yet-unconfirmed Csk1 targets such as Lsk1. Cdk1 requires T-loop phosphorylation for its essential function [58], precluding a simple, direct test of UV-sensitivity in a *cdc2^T167A^* mutant.

Also complicating matters is the likelihood that more than one DNA damage response pathway depends on CDKs, as suggested by the suppression of both conversion- and deletion-type recombination events in a *csk1Δ* strain (Fig. 4D). Csk1 and Rhp55/57 appear to act in independent pathways that converge on Rhp51 recombinase—a relationship similar to the one between Rhp55/57 and Swi5/Sfr1 [34]–[36]. By one possible interpretation, the complex UV-dose response of a *csk1 sfr1* double mutant (Fig. 3D) is consistent with both genes acting in the same pathway. In this scenario, deletion of *sfr1^+^* suppresses hypersensitivity of a *csk1Δ* strain at low doses because it favors efficient processing of HR intermediates by Rhp55/57, lessening the need for Csk1. At intermediate doses, *csk1Δ* is epistatic to *sfr1Δ*, perhaps because the Rhp55/57 pathway has been saturated. The synthetic phenotype at still higher doses might reflect additional defects due to impaired function by other CDK targets. Likewise, complex genetic interactions with *srs2*—epistasis at low, and synthetic hypersensitivity at higher doses—could be explained by Csk1 loss impinging on multiple pathways. Both Sfr1 and Srs2 contain sites matching the consensus sequence for phosphorylation by Cdk1, and Srs2 is a suspected Cdk1 target in budding yeast [59], [60], suggesting that Csk1 could act at least in part through the cell-cycle CDK to influence DNA damage repair.

## Materials and Methods

### General Fission Yeast Methods

Fission yeast cell culturing, transformation, sporulation and tetrad dissection were performed according to standard methods [61]. All relevant strains are listed in Table 1. Cells were grown in yeast extract medium with supplements (YES) unless stated otherwise. The *cdk9^T212E^* mutation was constructed and introduced into the genome as described elsewhere for *cdk9^T212A^* [20].

**Table 1:** Strains used in this study

Strain	Genotype	Source
JS78	*leu1-32 ura4-D18 his3-D1 ade6-M210 h+*	J Hurwitz
JS80	*csk1::ura4^+^ leu1-32 ura4-D18 his3-D1 ade6-M216 h+*	J Saiz
JS155	*csk1::kanMX leu1-32 ade6-M210 his3-D1 ura4-D18 h+*	J Saiz
JS167	*mcs6::mcs6 HA3/kanMX leu1-32 ade6-M216 his3-D1 ura4-D18 h-*	J Saiz
JS207	*mcs6:: mcs6^S165A^ HA3/kanMX4 leu1-32 ura4-D18 his3-D1 ade6-M216 h-*	J Saiz
YP1	*rad13::ura4^+^ leu1-32 ade6-704 ura4-D18 h-*	G Freyer
YP6	*rhp51::ura4^+^ ura4-D18 h+*	G Freyer
YP25	*rhp54::ura4^+^ ura4-D18 h+*	G Freyer
YP27	*rhp57::ura4^+^ ura4-D18 leu1-32 ade6-M216 h+*	G Freyer
YP46	*rad3::ura4^+^ leu1-32 ade6-704 ura4-D18 h-*	G Freyer
YP68	*csk1::kanMX rad3::ura4^+^ leu1-32 ura4-D18*	Y Pikman
YP85	*csk1::kanMX rad13::ura4^+^ leu1-32 ura4-D18*	Y Pikman
HD2-55	*csk1::kanMX rhp54:: ura4^+^ ura4-D18*	H Du
HD4-6	*csk1::kanMX rhp51::his3^+^ his3-D1*	H Du
HD7-24	*cdk9::cdk9^T212A^:kanMX6 leu1-32 ura4-D18 his3-D1 ade6-M210 h+*	H Du
HD7-44	*csk1::ura4^+^ cdk9::cdk9^T212A^:kanMX6 leu1-32 ura4-D18 his3-D1 ade6*	H Du
HG11	*leu1-32, ura4-D18, his3-D1, ade6-M375 int::pUC8/his3^+^/ade6-L469 h+*	M Whitby
HG16	*csk1::kanMX leu1-32, ura4-D18, his3-D1, ade6-M375 int::pUC8/his3^+^/ade6-L469*	This work
HG13	*srs2::ura4^+^, leu1-32, ura4-D18, his3-D1, ade6-M375 int::pUC8/his3^+^/ade6-L469 h+*	This work
HG19	*srs2::ura4^+^, csk1Δ::kanMX, leu1-32, ura4-D18, his3-D1, ade6-M375 int::pUC8/his3^+^/ade6-L469*	This work
HG24	*sfr1:: ura4^+^, leu1-32, ura4-D18, his3-D1, arg3-D1 Msmt-0*	H. Iwasaki
HG31	*sfr1:: ura4^+^, csk1Δ::kanMX, leu1-32, ura4-D18, his3-D1*	This work
HG112	*srs2::ura4^+^, csk1Δ::kanMX, leu1-32, ura4-D18, his3-D1, ade6-M21x*	This work
HG119	*srs2::ura4^+^, csk1Δ::kanMX, leu1-32, ura4-D18, his3-D1, ade6-M21x*	This work
HG123	*rhp57::ura4^+^, csk1Δ::kanMX^+^ ura4-D18 leu1-32 ade6-M216*	This work
HG127	*cdk9::cdk9^T212E^:kanMX, ade6-M210, leu1-32, ura4-D18, his3-D1 h+*	This work
HG133	*csk1::ura4^+^ cdk9::cdk9^T212E^ kanMX, ade6-M21X, leu1-32, ura4-D18, his3-D1*	This work
HG142	*csk1::kanMX leu1-32, ura4-D18, his3-D1, ade6-M375 int::pUC8/his3^+^/ade6-L469*	This work
HG144	*csk1::kanMX leu1-32, ura4-D18, his3-D1, ade6-M375 int::pUC8/his3^+^/ade6-L469*	This work

### DNA damage sensitivity tests

To create UV and IR survival curves, cells were grown to early-mid log phase, plated and irradiated using either a 254 nm UV light source or ^60^Co gamma-ray source, respectively, with different doses. Colonies were counted after 3–5 days. Percent survival was as compared to colony counts after cells were plated but not irradiated. For spot assays, 10-fold serial dilutions of cells in mid-log phase were plated on media containing MMS, HU or no drug and incubated 3–5 days before photographing.

### Checkpoint assay

To synchronize cells in G2, 25-ml cultures were grown in YES to mid-log phase, pelleted, resuspended in 750 µl YES, and layered on top of a 15-ml 7–30% lactose gradient. The gradient was spun at 1000 rpm for 8 min and a 500-µl G2 portion of the gradient was removed. The cells were plated on YES, half were exposed to 40 J/m^2^ UV light and recultured in 5 ml YES. Samples were fixed in methanol at indicated timepoints, stained with DAPI and calcofluor, and 200 cells per time point were scored for passage through mitosis (binucleated, septated and doublet cells were counted as having passed mitosis).

### Recombination frequency measurements

Spontaneous recombination frequencies were measured by measuring numbers of *ade^+^* colonies arising from a strain containing two *ade6* heteroalleles flanking a *his^+^* gene [37]. Cells were first plated onto minimal media lacking histidine and containing only 9.75 µg/ml adenine to confirm that recombination had not yet occurred between the *ade6* alleles. Three red (i.e., *ade-*) colonies were then plated on YES and grown for 4 days. Five colonies from each of the three plates were resuspended in water, plated on media lacking adenine to determine the total frequency of *ade6^+^* colonies, and then replica-printed on media lacking histidine to determine the percent of *ade6^+^* colonies that lost the *his^+^* gene. Frequencies from the five colonies were averaged and the final value was determined from the mean of frequencies resulting from the three colonies. Error bars indicate standard deviation from the mean in each experiment.

Csk1 was overexpressed in JS80 from an ADH promotor in the plasmid pHG2, which is marked with a *kanMX* drug-resistance marker, and transformants were selected for growth on YES media containing 200 µg/ml G418.
